# Heterocellular induction of interferon by negative-sense RNA viruses

**DOI:** 10.1016/j.virol.2010.08.008

**Published:** 2010-11-25

**Authors:** S. Chen, J.A.L. Short, D.F. Young, M.J. Killip, M. Schneider, S. Goodbourn, R.E. Randall

**Affiliations:** a School of Biology, Centre for Biomolecular Sciences, BMS Building, North Haugh, University of St. Andrews, St. Andrews, Fife, KY16 9ST, UK; b Division of Basic Medical Sciences, St. George's, University of London, London SW17 0RE, UK

**Keywords:** Paramyxovirus, Influenza bunyamwera, Interferon

## Abstract

The infection of cells by RNA viruses is associated with the recognition of virus PAMPs (pathogen-associated molecular patterns) and the production of type I interferon (IFN). To counter this, most, if not all, RNA viruses encode antagonists of the IFN system. Here we present data on the dynamics of IFN production and response during developing infections by paramyxoviruses, influenza A virus and bunyamwera virus. We show that only a limited number of infected cells are responsible for the production of IFN, and that this heterocellular production is a feature of the infecting virus as opposed to an intrinsic property of the cells.

## Introduction

Virus infection leads to rapid activation of the innate immune response of the host that is associated with a short-term restriction of virus replication. One of the main components of the innate anti-viral response is type I interferon (IFN), a group of closely-related cytokines. Type I IFNs signal through a common receptor to up-regulate over 400 genes (the so-called IFN-stimulated genes, or ISGs) by activating the well-characterised JAK/STAT pathway (Platanias, 2005; Randall and Goodbourn, 2008). Many of the ISGs, including protein kinase R (PKR), 2′5′ oligoadenylate synthetase (OAS) and MxA have anti-viral activity, and thereby act to limit the spread of the virus (Sen and Peters, 2007).

IFN induction requires the recognition of pathogen-associated molecular patterns (PAMPs), which are seen as foreign by pattern recognition receptors (PRRs, reviewed in Takeuchi and Akira, 2009). Two well-characterised cytoplasmic PRRs, retinoic acid inducible gene I (RIG-I), and melanoma differentiation-associated gene-5 (mda-5) are thought to recognise respectively blunt short double-stranded 5′-triphosphorylated RNA or long dsRNA (Kato et al., 2008; Schlee et al., 2009; Schmidt et al., 2009), and are thus of considerable importance in mediating IFN induction by RNA viruses (reviewed in Wilkins and Gale).

To survive in nature all viruses appear to require a strategy to circumvent the IFN response. The evasion strategies can be classified as (i) generally inhibiting cellular transcription and/or protein synthesis, (ii) specifically inhibiting components of the IFN-induction or IFN-signalling pathways, or (iii) inhibiting IFN-induced factors that have anti-viral activity (Randall and Goodbourn, 2008). The nature of IFN antagonists encoded by small negative-sense RNA viruses reflects these processes. As examples, the NSs protein of bunyamwera virus (BUNV) inhibits cellular transcription, thereby inhibiting both the production of IFN and the ability of infected cells to respond to IFN (Thomas et al., 2004); the NS1 protein of influenza A viruses (FLUAV) is a multifunctional protein that specifically inhibits RIG-I activation, generally interferes with cellular protein expression by inhibiting the correct processing and export of cellular mRNAs from the nucleus to cytoplasm, and blocks the activity of PKR and OAS (Hale et al., 2008). The IFN antagonists of paramyxoviruses specifically block both IFN induction and IFN signalling, although the exact molecular mechanisms by which they achieve this vary between different viruses (Fontana et al., 2008; Goodbourn and Randall, 2009; Ramachandran and Horvath, 2009); thus, the V protein of parainfluenza virus type 5 (PIV5) blocks IFN signalling by catalytically targeting STAT1 for proteasome-mediated degradation (Didcock et al., 1999; Precious et al., 2005, 2007), whereas the V protein of parainfluenza virus type 2 (PIV2) targets STAT2 for degradation (Parisien et al., 2001). The V proteins of paramyxoviruses block IFN induction (Poole et al., 2002; He et al., 2002) by binding to mda-5 (Andrejeva et al., 2004; Childs et al., 2007) and preventing its dimerisation (Childs et al., 2009), whilst the C protein of Sendai virus (SeV) blocks IFN induction through RIG-I (Strahle et al., 2007).

Despite the existence of potent virus-encoded antagonists of the IFN system, IFN can still exert an anti-viral effect that limits the production of virus. This is supported by the observation that RNA viruses produce larger plaques on cells that have been engineered to either fail to produce or respond to IFN than they do on unmodified ‘IFN-competent’ cells (Young et al., 2003). The apparent incomplete block to the IFN system suggests that the spread of a virus through a cell population is a complex process. To better understand how viruses interact with the IFN response we have been following the development of PIV5 plaques in cells that can produce and respond to IFN (Carlos et al., 2009). Under these conditions the virus continues to slowly spread from cell-to-cell despite inducing limited amounts of IFN. It achieves this by infecting cells in a pre-existing IFN-induced anti-viral state and targeting STAT1 for degradation (Didcock et al., 1999; Precious et al., 2007). In the absence of continuous IFN signalling the cell cannot maintain its anti-viral state indefinitely and eventually normal virus replication is established (Carlos et al., 2009; Precious et al., 2007). To further build up a picture of the factors which govern how PIV5, and by extension other paramyxoviruses, spread through cells which can produce and respond to IFN, we have studied the dynamics of IFN production by cells within developing PIV5 plaques. We present evidence that there is heterocellular induction of the IFN-β promoter within PIV5 infected cells, thus suggesting that only a few infected cells within developing plaques are responsible for IFN that is produced. Similarly, evidence is presented that only a few infected cells within developing plaques of other members of the paramyxovirus family, as well as with influenza A virus (strain Udorn) and bunyamwera virus, produce IFN, suggesting that heterocellular production of IFN is a general consequence of infection with negative strand RNA viruses.

## Results

### The establishment of an anti-viral state at the site of a developing PIV5 infection

**Fig. 1:**
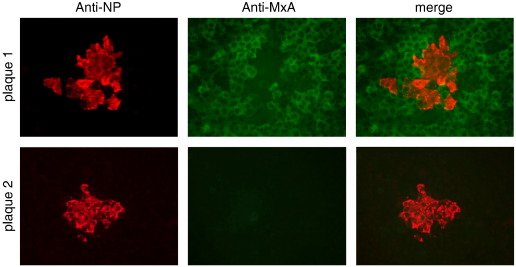
At two days p.i., the uninfected cells surrounding developing plaques may (plaque 1), or may not (plaque 2), be positive for MxA. Monolayers of A549 cells grown on coverslips were infected at an moi of 0.001 pfu/cell. At 48 h p.i. the cells were fixed, stained with anti-NP and anti-MxA antibodies, and visualised using a Nikon Microphot-FXA immunofluorescence microscope.

To follow the induction of an IFN-induced anti-viral state in cells surrounding sites of infection, A549 cells were seeded onto coverslips and infected with PIV5 at an moi of 0.001 pfu per cell (i.e. under these conditions most of the cells would not be infected at early time points). At various times post-infection (p.i.), the cells were fixed, and infected cells identified by immunofluorescence using an antibody directed against the nucleoprotein (NP) of PIV5. The cell population was also co-stained for MxA, which is an anti-viral protein induced in uninfected cells as a consequence of IFN binding to the type I IFN receptor. Two days after infection we routinely saw groups of 10–30 cells expressing viral antigen, indicative of viral replication and spread. Surprisingly, only some of these groups of cells were surrounded by a layer of cells that were positive for MxA (Fig. 1). Continued monitoring of the size of groups of infected cells showed that by 4 days p.i. all the remaining uninfected cells within the infected monolayers were positive for MxA, demonstrating that enough IFN had been secreted into the media to induce an anti-viral state in all the uninfected cells (data not shown). From these initial observations we concluded that by two days p.i., although some infected cells must have produced IFN, this was not the case for all infected cells.

### Generation and characterisation of an A549/pr(IFN-β). GFP reporter cell line

**Fig. 2:**
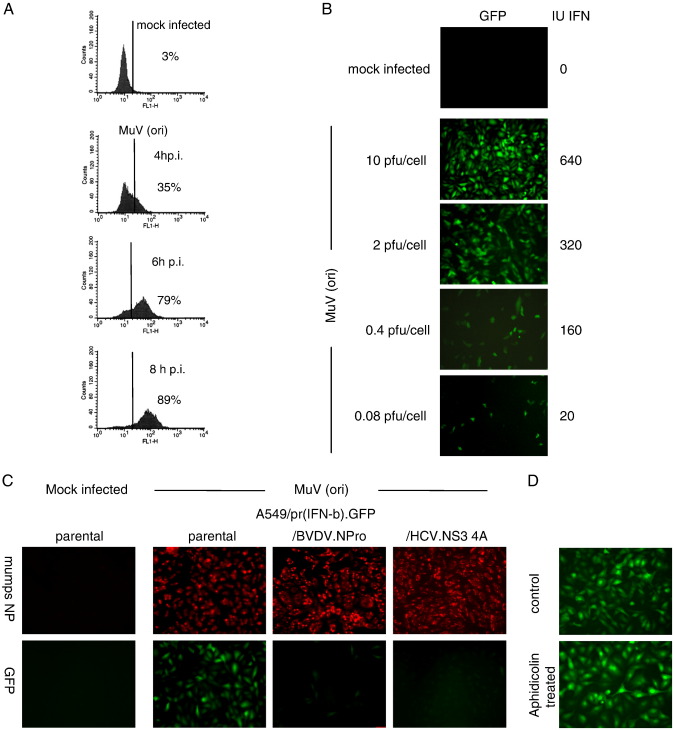
Characterisation of the A549/pr(IFN-β).GFP reporter cells. (A). Monolayers of A549 cells were either mock-infected or infected with 5 pfu per cell of a stock of MuV (ori) known to be a good inducer of IFN; at 4, 6 and 8 h p.i. the cells were trypsinised to a single cell suspension and the percentage of GFP-positive cells estimated by FACS analysis. (B). Confluent monolayers of A549/pr(IFN-β).GFP cells were grown in 60 mm dishes that contained coverslips and infected at various multiplicities of infection of MuV(ori). At 12 h p.i. the coverslips were fixed and those cells expressing GFP visualised using a Nikon Microphot-FXA fluorescence microscopy. In addition, the amount of IFN secreted into the culture medium was determined (right hand column). (C). A549/pr(IFN-β).GFP, A549/pr(IFN-β).GFP/BVBV.N^pro^ or A549/pr(IFN-β).GFP/HCV.NS34a cells were either mock-infected of infected with MuV(ori) at 5 pfu/cell. At 12 h p.i. the cells were fixed and immunostained with an anti-NP mAb. GFP-positive and virus-infected cells were visualised by fluorescence microscopy. (D). A549/pr(IFN-β).GFP were, or were not (control), treated with aphidicolin (1 μg/ml) for 16 h, infected with MuV (ori) at 5 pfu/cell in the presence or absence of aphidicolin as appropriate, fixed at 16 h p.i. and GFP-positive cells visualised by fluorescence microscopy.

The heterogeneity of the MxA induction seen in the above experiment could result from either heterogeneity or temporal lags in the production or response of IFN. To distinguish between these possibilities we generated an A549 reporter cell line in which IFN production was monitored by placing the eGFP gene under the control of the IFN-β promoter. To generate such a cell line, the first 282 bp of the IFN-β promoter were cloned into a bicistronic lentivirus vector, generating a plasmid (pdl′pIFNβ′GFP) such that following activation of the IFN-β promoter both eGFP and pac (which confers puromycin resistance) are expressed. Following transduction of A549 cells with the vector, cells were first subjected to puromycin selection for 2 days following stimulation with dsRNA for 8 h. Subsequently surviving cells were transfected with dsRNA for 8 h and GFP-positive single cells separated by FACS into individual wells of a 96-well microtitre plate such that a colony appeared in approximately 10–20% of wells. The resulting colonies were screened for their ability to express GFP in response to infection with a stock of MuV that we knew to be an extremely good inducer of IFN as it contained large amounts of defective interfering particles (DIs) that are powerful activators of the IFN-induction cascade (this stock will be referred to hereafter as MuV[ori]). The best colony of responding cells (termed A549/pr(IFN-β).GFP) in terms of numbers of positive cells and intensity of GFP expression was then further characterised. Fig. 2A shows that by 4 h p.i. approximately 35% of cells were positive for GFP expression, by 6 h p.i. 80% were positive and by 8 h p.i. 90% of the cells were strongly positive upon infection with MuV(ori). FACS analysis of the MuV(ori) infected cells showed a discrete population of GFP-positive cells rather than a gradient of GFP-positive cells, suggesting that the IFN-β promoter is either ‘on’ or ‘off’ in infected cells. Next, A549/pr(IFN-β).GFP cells that had been grown on coverslips in 24-well Linbro plates were infected with differing multiplicities of MuV(ori). At 24 h p.i. the number of cells expressing GFP was visualised by fluorescence and the amount of IFN in the culture medium estimated using a CPE-reduction bio-assay (Fig. 2B), showing that the number of cells expressing GFP correlated with the amount of IFN produced.

To ensure expression of GFP was being activated by the well-characterised IFN-induction pathways, we also determined the effect of knocking out key signalling molecules involved in the IFN-induction cascade. To this end we engineered the A549/pr(IFN-β).GFP reporter cell line to either constitutively express the NS3/4a protein of HCV, which proteolytically cleaves the adaptor molecule CARDIF/MAVS/IPS1/VISA, or the N^pro^ protein of BVDV, which targets IRF-3 for proteasome-mediated degradation. As expected, expression of either HCV NS3/4a or BVDV-N^pro^ prevented induction of GFP expression following infection of the cells with MuV (Fig. 2C).

Having successfully isolated a cell line in which GFP expression was under the control of the IFN-β promoter we performed a series of additional experiments to further characterise the cell line. Given that the cells have been engineered such that they have at least one additional IFN-β promoter it is theoretically possible that there is competition between the inserted promoter and the natural promoter. If this were the case it would be expected that the amount of IFN secreted by the reporter cells would be less than that secreted by the parental cell line. However, both cell lines produced similar amounts of IFN when infected (data not shown). To determine whether GFP expression was transient following activation of the IFN-β promoter live cell microscopy was used to follow the fate of individual cells that became positive for GFP expression over a 48 h period. These studies demonstrated that once a cell had become positive for GFP expression it remained positive (data not shown). Finally, it has previously been suggested that the induction of the IFN-β promoter may be dependent upon the cell cycle (Zawatzky et al., 1985). Whilst this seems unlikely from the observation that > 80% of A549/pr(IFN-β).GFP cells growing asynchronously were positive for GFP following infection with MuV (ori), we tested this directly by arresting cells at the G1-S boundary using aphidicolin prior to infection with MuV(ori). No difference was observed between the number of GFP-positive cells that had or had not been treated with aphidicolin demonstrating that the cell cycle does not influence the activation of the IFN-β promoter in these reporter cell lines (Fig. 2D).

**Fig. 3:**
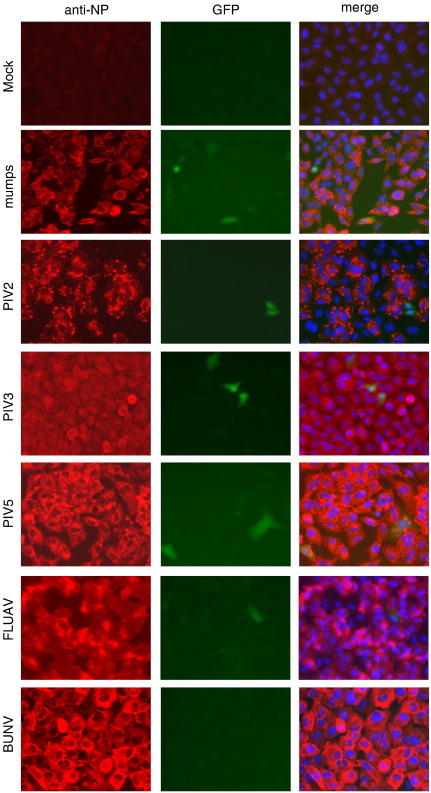
Detection of GFP-positive cells following infection of A549/pr(IFN-β).GFP cells with a panel of negative strand RNA viruses. A549/pr(IFN-β).GFP cells were infected with MuV (cl3), PIV2, PIV3, PIV5, FLUAV or BUNV at 2–5 pfu/cell. At 16 h p.i. the cells were fixed and immunostained with an anti-NP mAb. GFP-positive and virus-infected cells were visualised by fluorescence microscopy. The presence of the nuclei in the merge images was visualised by DAPI staining. Note that MuV (cl3/30) was originally plaque-purified from MuV (ori).

The data above show that efficient inducers of IFN-β (i.e. a MuV preparation rich in DI particles, or synthetic PAMPs; data not shown) cause a robust induction of GFP expression in the majority of A549/pr(IFN-β).GFP cells, with responsive cells producing similar levels of fluorescence. We next used the A549/pr(IFN-β).GFP cells to monitor the activation of the IFN-β promoter in individual cells during infection by viruses generated by passage at low m.o.i. Such preparations would be typical working stocks, lacking or low in DI particles, and are generally poor inducers of IFN-β (see for example Poole et al., 2002). Strikingly, in contrast to the extensive induction seen by the DI-rich preparation, MuV(ori) — see Fig. 2, a plaque-purified preparation of MuV, termed MuV cl3/30 (Young et al., 2009), showed high infectivity but only generated a few GFP-positive cells (Fig. 3). Each of the GFP-positive cells showed a similar degree of fluorescence, as described above for the MuV(ori) infection.

**Fig. 4:**
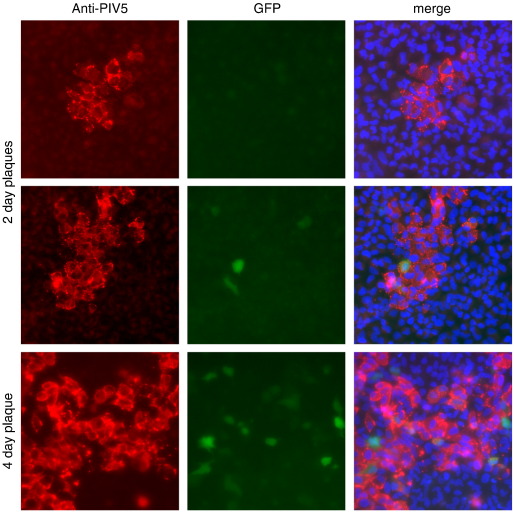
Detection of GFP-positive cells within developing plaques of PIV5. A549/pr(IFN-β).GFP cells grown on coverslips were infected at an moi of 0.001 pfu/cell. At 2 and 4 days p.i. the cells were fixed and immunostained with an anti-NP, and GFP-positive cells and infected cells visualised by fluorescence microscopy. Note that at two days p.i. there were a mixture of plaques which did, or did not, contain GFP-positive cells. By 4 days p.i. GFP-positive cells could be detected in all plaques, although the majority of infected cells still remained negative for GFP. The presence of the nuclei in the merge images was visualised by DAPI staining.

**Fig. 5:**
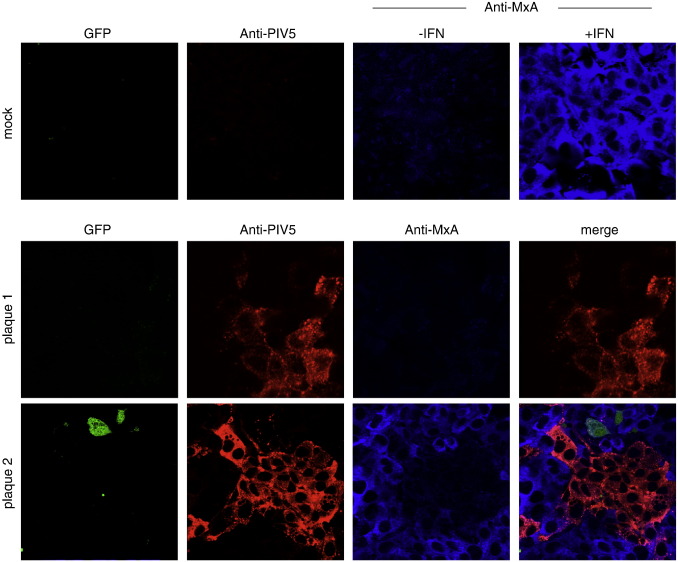
At two days p.i. there are a mixture of PIV5 plaques in which the IFN-β promoter has, or has not, been activated in some infected cells. A549/pr(IFN-β). GFP cells grown on coverslips were mock-infected, or were infected at an moi of 0.001 pfu/cell. At 2 days p.i. the cells were fixed and immunostained with anti-NP and anti-MxA antibodies. GFP-positive cells (green), MxA-positive cells (blue) and infected cells (red) were visualised by confocal microscopy. To show that MxA is induced by IFN, its cytoplasmic distribution, and specificity of the antibody, mock-infected cells were (+ IFN) or were not (−IFN) treated with IFN for 20 h and immunostained as above. Note that expression of GFP correlated with whether the uninfected cells surrounding the plaque were positive for MxA; if a plaque contained a GFP-positive cell the surrounding cells were positive for MxA, if not they were negative.

To investigate whether our observations on MuV were typical for other RNA viruses we tested the paramyxoviruses PIV2, PIV3 and PIV5, and the other negative-stranded RNA viruses, influenza A virus (FLUAV), and bunyamwera virus (BUNV). Each of these viruses gave similar results to plaque-purified MuV, i.e. efficient infection of cells, but a heterocellular induction of GFP (Fig. 3). Heterocellular induction of IFN-β might account for the heterogeneity of the MxA induction seen during PIV5 infection (see Fig. 1). To examine this directly we used the A549/pr(IFN-β).GFP cells to monitor the activation of the IFN-β promoter in individual cells within a developing plaque. A549/pr(IFN-β).GFP reporter cells were infected with PIV5 at an moi of 0.001 pfu/cell and the expression of GFP monitored various times post-infection. These results showed that GFP was expressed in only a minority of infected cells within the developing plaques. At 2 days p.i. there was a mixture of plaques in which either no cells were positive for GFP expression, or in which only one or two GFP-positive cells could be detected. However, by 4 days p.i. some GFP-positive cells could be detected in all plaques (Fig. 4). The absence of GFP-positive cells within some 2 day-old plaques suggested that the IFN-β promoter had not been activated, and consequently IFN would not have been secreted, by any of the cells within these plaques. To determine if this was the case, we set up a similar experiment, but also stained the monolayers for MxA as a marker for IFN activity (Fig. 5). Consistent with the data shown in Fig. 1, at two days p.i. there were some plaques in which none of the surrounding cells were positive for MxA, whilst around other plaques the uninfected cells were positive for MxA. Strikingly, expression of MxA directly correlated with the presence of a GFP-positive cell within the developing plaques. If no GFP-positive cells were detected, then the surrounding uninfected cells were negative for MxA, whilst if there were one or more GFP-positive cells within a plaque then the surrounding cells were MxA-positive. [Note that the cells in the centre of plaque 2 in Fig. 5 are negative for MxA, presumably because in these cells, the first to be infected, the virus had targeted STAT1 for degradation and thus the cells could not respond to IFN once it was secreted by the GFP-positive cell.]

**Fig. 6:**
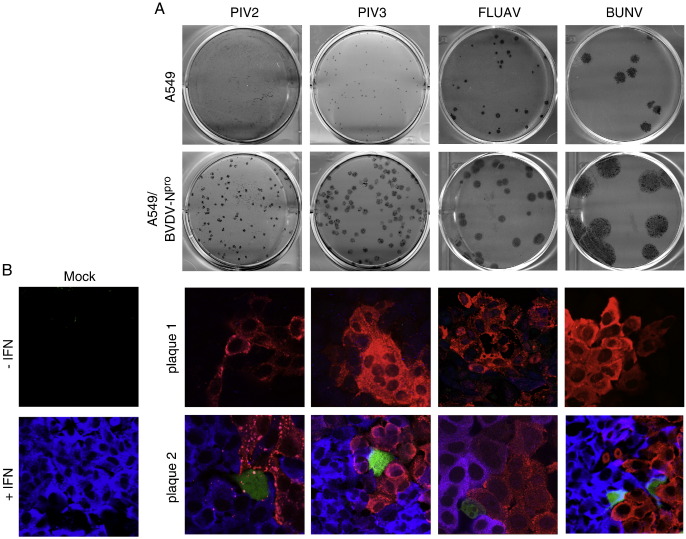
Heterocellular induction of the IFN-β promoter in developing plaques of PIV2, PIV3, FLUAV and BUNV. (A) PIV2, PIV3, FLUAV and BUNV form larger plaques on A549/BVDV-N^pro^ cells (that cannot to produce IFN) compared to parental A549 cells (which can produce and respond to IFN). (B) At 2 days p.i. some, but not all, PIV2, PIV3, FLUAV and BUNV plaques contain a few cells in which the IFN-β promoter has been activated. A549/pr(IFN-β).GFP cells grown on coverslips were infected at an moi of 0.001 pfu/cell. At 2 days p.i. the cells were fixed and immunostained with anti-NP and anti-MxA antibodies. GFP-positive cells (green), MxA-positive cells (blue/purple) and infected cells (red) were visualised by fluorescence microscopy. The presence of the nuclei in the merge images was visualised by DAPI staining. Note that expression of GFP always correlated with whether the uninfected cells surrounding the plaque were positive for MxA; if a plaque contained a GFP-positive cell the surrounding cells were positive for MxA, if not they were negative.

We also examined whether the same effects were seen during the course of other RNA virus infections. Each of PIV2, PIV3, FLUAV and BUNV produce larger plaques on cells that have been engineered to either not produce or respond to IFN (Fig. 6A), and thus some cells must be secreting IFN that is inducing an anti-viral state in the surrounding uninfected cells, thereby slowing the spread of the infection. When the production of IFN was monitored using the GFP reporter in A549/pr(IFN-β).GFP cells we observed that as seen for PIV5, the cells surrounding infected cells in plaques in which no GFP-positive cells could be detected, were negative for MxA expression (Fig. 6B), suggesting that no cells within these plaques had yet been activated to produce IFN. In contrast, the uninfected cells surrounding plaques containing at least one GFP-positive cell were positive for MxA (Fig. 6B). The fact that for all the viruses studied induction of MxA correlated with the presence of at least one GFP-positive cell within the developing plaque confirmed our previous conclusions that expression of GFP was a reliable marker to identify cells that were likely to have produced IFN. It should be noted that whilst the IFN-β promoter must be activated to induce GFP expression, the lack of GFP expression in infected cells could also be a consequence of post-transcriptional regulation brought about by viral antagonists such as the NS1 protein of FLUAV interfering with the processing and export of cellular mRNAs from the nucleus to the cytoplasm. Nevertheless, these results strongly suggest that only a limited number of cells infected with negative-sense RNA viruses produce the IFN that establishes an anti-viral state in the uninfected cells surrounding the developing plaque, thereby slowing the spread of the infection.

## Discussion

We have previously shown that when PIV5 infects cells in an IFN-induced anti-viral state there are very significant changes to the pattern of virus protein synthesis and cytoplasmic bodies containing the NP, P and L proteins (Carlos et al., 2005) and virus genomes become evident (Carlos et al., 2009). This IFN-induced anti-viral state is extremely effective at reducing virus replication. However, PIV5 is not eliminated from these cells but rather is able to dismantle the anti-viral state in the absence of continuing IFN signalling, by targeting STAT1 for proteolytic degradation (Precious et al., 2007); complete destruction of STAT1 is a prelude to a recommencing of normal PIV5 replication with the eventual release of virus. Nevertheless, the potential of IFN to significantly slow the spread of virus presents a formidable obstacle, and thus PIV5 has evolved a potent mechanism(s) to limit IFN production (Andrejeva et al., 2004; Childs et al., 2009; He et al., 2002; Poole et al., 2002). These mechanisms are not fully effective however, as demonstrated by the fact that PIV5 (like other negative strand RNA viruses) produces larger plaques in ‘IFN-compromised’ cells than in ‘IFN-competent’ cells — i.e. at some point at least some virus-infected cells must have secreted IFN thereby slowing the spread of infection (data presented here, and Young et al., 2003).

Whilst studying the dynamics of IFN production relative to IFN responses during an ongoing PIV5 infection we observed that at 2 days p.i., uninfected cells surrounding some developing plaques did not express MxA and thus could not have been in an IFN-induced anti-viral state (unpublished observations, Fig. 1). From this we concluded that it was unlikely that IFN was synthesised and secreted from all infected cells as otherwise the uninfected cells surrounding all the plaques should have been positive for MxA. To further study the dynamics of virus:host cell interactions that lead to the activation of the IFN system, we generated a cell line (A549/pr(IFN-β).GFP) in which the expression of GFP was under the control of the IFN-β promoter. Most (at least 90%), if not all, of the cells in this population are able to respond rapidly to efficient inducers of IFN, in a manner that depends upon the canonical signalling pathways. Having established that GFP expression was a reliable marker to identify individual cells in which the IFN-β promoter has been activated we subsequently used the A549/pr(IFN-β).GFP reporter cells to follow the induction of GFP in individual cells infected at a high moi by PIV5, and also PIV2, PIV3, BUNV and FLUAV. These results clearly demonstrated that GFP was only induced in a small minority of infected cells. We also investigated the induction of GFP in individual cells within developing plaques of these viruses. At two days p.i. some plaques were readily visualised which contained no GFP-positive cells, suggesting that IFN had not been produced in any of the infected cells within these plaques. This conclusion was supported by the observation that none of the uninfected cells surrounding plaques in which there were no GFP-positive cells were positive for MxA. In contrast, other plaques could also be detected in which a few of the infected cells were positive for GFP and, furthermore, the uninfected cells surrounding these plaques were always positive for MxA. Taken together, these results strongly suggest that only a few cells within developing plaques of negative-sense RNA viruses produce the IFN that is responsible for inducing the anti-viral state in the surrounding uninfected cells that reduces the speed of virus spread and consequently plaque development.

It has been previously reported that the IFN-β gene shows heterocellular induction in response to either the synthetic dsRNA, poly(I).poly(C), or Sendai virus (Apostolou and Thanos, 2008; Enoch et al., 1986; Hu et al., 2007; Senger et al., 2000; Zawatzky et al., 1985). In none of these cases has the molecular basis of the restriction of induction been determined, but it has been generally assumed to be a property of the host cell; for example, it has been suggested that only cells at certain stages of the cell cycle may be responsive (Zawatzky et al., 1985). More recently, it has been demonstrated that the percentage of cells able to support induction can be lowered by overexpressing IRF-2, an antagonist of IFN-β transcription (Senger et al., 2000), or raised by overexpressing the IFN-β transcriptional activators p65 and/or IRF-3 (Apostolou and Thanos, 2008), suggesting that the restriction is a result of the limited availability of transcription factors. However, in our experiments, given that GFP can be induced in at least 90% of the A549/pr(IFN-β).GFP cells (even in cells which have been blocked in their cell cycle), and that the reporter gene does not compete with the endogenous IFN-β gene, it clearly indicates that induction in these cells is not restricted by transcription factor availability or signalling pathway activation. Therefore, the heterocellular induction observed in the A549/pr(IFN-β).GFP cells must be due to the property of the infecting virus, rather than the ability of the cells to respond to virus infection. Whilst variability in the infecting virus population was not considered as a possible cause of the observed heterocellular induction of the IFN-β promoter in most of the previous studies, it remains possible that different cell types might show very different percentages of cells able to support IFN-β induction, with some cell lines, including A549 cells, able to induce IFN in nearly every cell in a population.

It is well-established that most, if not all, viruses encode antagonists of the IFN system, especially antagonists that limit IFN induction (reviewed by Haller et al., 2006; Randall and Goodbourn, 2008). Given that the heterocellular induction of GFP is clearly a property of the infecting virus, a critical question to address is why GFP, and thus IFN, induced in some cells but not others. One possible explanation is that the cells that become GFP-positive represent those cells in which the virus has failed to block the signal transduction pathways activated by viral PAMPs produced during normal virus replication, or has globally failed to inhibit cellular transcription and or protein synthesis. An alternative possibility is that the positive cells contain virus infections that have either overproduced PAMPs, or have perhaps produced a corrupt PAMP, such as that associated with DI particles, which the virus-encoded IFN antagonists cannot block. Regardless of the explanation for heterocellular induction of the IFN-β promoter, it is clear from the approach taken here that there is still much to be learnt about the processes and dynamics by which viruses activate the IFN response and some of these questions will only be answered by examining the activation of the IFN-β promoter in individual cells. The A549/pr(IFN-β).GFP reporter cells, their derivatives and/or equivalents, should prove to be invaluable tools in such studies.

## Materials and methods

### Cells, viruses and plasmids

Vero, MDCK and A549 cells, and their derivatives, were grown as monolayers in Dulbecco's modified Eagle's medium (DMEM) supplemented with 10% (growth medium) or 2% (maintenance) foetal bovine serum at 37 °C. PIV5 (strain W3A; Choppin, 1964), PIV2, PIV3, MuV (strain Enders) and BUNV were grown and titrated under appropriate conditions in Vero cells, or where stated on A549 cells. FLUAV (strain Udorn) was grown and titrated on MDCK cells, or where stated on A549 cells, in the presence of 2 μg/ml N-acteyl trypsin. The construction and properties of cell lines expressing BVDV-N^pro^ have been previously described (Hilton et al., 2006) and hepatitis C virus (HCV) NS3/4a-expressing cells were generated in a similar manner. Briefly, the NS3/4a gene was cloned into a modified self-inactivating, bicistronic, lentiviral expression vector derived from pHR-SIN-CSGW (Demaison et al., 2002), and used to generate recombinant lentivirus particles that were used to select for cells that express V5-tagged NS3/4a as previously described (Hilton et al., 2006).

### Immunofluorescence, FACS and immunoblot analysis

The procedures for immunoblotting and immunofluorescence have previously been described (Carlos et al., 2005; Randall and Dinwoodie, 1986). Antibodies used in these procedures included monoclonal antibodies (mAbs) to the nucleoproteins of PIV2 (PIV2-NPa, Randall and Young, 1988), PIV3 (4481, 4721 and 4812, a kind gift from Claes Orvell, Karolinska Universitetssjukhuset, Sweden, Rydbeck et al., 1986), PIV5 (PIV5-NPa, Randall et al., 1987), FLUAV (abcam, ab20343) and a mAb to MxA (a kind gift from Otto Haller, University of Freiburg, Germany, Flohr et al., 1999). Also used was a polyclonal antibody to the NP of BUNV (a kind gift from Richard Elliott, University of St Andrews). Following immunostaining, monolayers were washed with PBS, mounted using either Citifluor AF-1 mounting solution (Citifluor Ltd., UK) or Mowiol and examined with either a Nikon Microphot-FXA immunofluorescence microscope or a Zeiss LSM 5 Exciter confocal microscope. For FACS analysis, cells were trypsinised to a single cell suspension, fixed and permeabilised as for immunofluorescence, and immunostained with the mAbs to the NP of MuV. The percentage of fluorescent cells, and intensity of their fluorescence in 10,000 events was determined by using the LYSYS programme on a Becton Dickinson FACScan.

### Interferon and interferon assays

Cells were treated with IFN-α (Roferon A, Roche) at 1000 U/ml. The amount of IFN secreted by cells was determined by a CPE-reduction bio-assay. Briefly, culture supernatants from infected cells were harvested, centrifuged at 1500 × *g* for 10 min to pellet cellular debris, UV-treated to inactivate residual virus, then serially diluted 2-fold and added to A549/BVDV-N^pro^ cell monolayers for 18 h prior to infection with EMCV (0.05 pfu/cell). Monolayers were fixed 2–3 days post-infection (with PBS + 5% formaldehyde) and cytopathic effect (CPE) monitored by staining with 0.1% crystal violet.
